# Towards Patient-Specific Computational Modelling of Articular Cartilage on the Basis of Advanced Multiparametric MRI Techniques

**DOI:** 10.1038/s41598-019-43389-y

**Published:** 2019-05-09

**Authors:** Kevin Linka, Amelie Schäfer, Markus Hillgärtner, Mikhail Itskov, Matthias Knobe, Christiane Kuhl, Lea Hitpass, Daniel Truhn, Johannes Thuering, Sven Nebelung

**Affiliations:** 1 RWTH Aachen University, Department of Continuum Mechanics, 52072 Aachen, Germany; 2 Stanford University, Department of Mechanical Engineering, Stanford, CA 94305 USA; 3 Aachen University Hospital, Department of Orthopaedic Trauma, 52074 Aachen, Germany; 4 Aachen University Hospital, Department of Diagnostic and Interventional Radiology, 52074 Aachen, Germany; 5 RWTH Aachen University, Institute of Imaging and Computer Vision, 52074 Aachen, Germany

**Keywords:** Medical research, Biomedical engineering

## Abstract

Cartilage degeneration is associated with tissue softening and represents the hallmark change of osteoarthritis. Advanced quantitative Magnetic Resonance Imaging (qMRI) techniques allow the assessment of subtle tissue changes not only of structure and morphology but also of composition. Yet, the relation between qMRI parameters on the one hand and microstructure, composition and the resulting functional tissue properties on the other hand remain to be defined. To this end, a Finite-Element framework was developed based on an anisotropic constitutive model of cartilage informed by sample-specific multiparametric qMRI maps, obtained for eight osteochondral samples on a clinical 3.0 T MRI scanner. For reference, the same samples were subjected to confined compression tests to evaluate stiffness and compressibility. Moreover, the Mankin score as an indicator of histological tissue degeneration was determined. The constitutive model was optimized against the resulting stress responses and informed solely by the sample-specific qMRI parameter maps. Thereby, the biomechanical properties of individual samples could be captured with good-to-excellent accuracy (mean *R*^2^ [square of Pearson’s correlation coefficient]: 0.966, range [min, max]: 0.904, 0.993; mean Ω [relative approximated error]: 33%, range [min, max]: 20%, 47%). Thus, advanced qMRI techniques may be complemented by the developed computational model of cartilage to comprehensively evaluate the functional dimension of non-invasively obtained imaging biomarkers. Thereby, cartilage degeneration can be perspectively evaluated in the context of imaging and biomechanics.

## Introduction

In the context of personalized medicine, biomechanical computational modelling becomes ever more relevant in patient-specific care^1^. Modelling-based predictions of biomechanical tissue properties hold great potential for the non-invasive and non-destructive characterization of the tissue status in health and disease; however, these go along with high requirements for simulation and measurement techniques. In particular, the constitutive model has to address significant variations in mechanical properties associated with age, gender, lifestyle as well as disease^2^. In the field of cartilage modeling, patient-specific predictions could significantly facilitate the detection of cartilage degeneration, which is the hallmark change of osteoarthritis (OA). Detection of early changes of particular relevance as the degenerative cascade may at least be slowed (if not even halted) in its progression if early preventive action such as modification of activity level, weight loss, pharmacological chondroprotection or axis-modifying surgery is taken in a timely manner^3^. However, up to now, it is not possible to detect the early, potentially reversible stages of cartilage degeneration using clinical standard imaging modalities^4,5^. Against this background, the non-invasive imaging of cartilage by advanced MRI techniques has made considerable progress over the last decades. Functional MRI techniques (synonymous with quantitative MRI [qMRI]) such as T2, T2* and T1*ρ* mapping have been developed and validated in a variety of scientific and clinical contexts to characterize extracellular matrix properties of cartilage^6–8^ and provide measures related to tissue composition and structure^4,9^. T2 relaxation describes the decay of transverse magnetization and is reflective of the tissue’s collagen structure and water content. In addition to the tissue-characteristic T2 relaxation, T2* relaxation is governed by additional T2* decay secondary to static magnetic field non-uniformity. In contrast, T1*ρ* relaxation is determined by measuring the decay of locked transverse magnetization and commonly considered indicative of low-frequency interactions between the tissue’s macromolecules and extracellular water. Meanwhile, the exact sensitivity and specificity profiles of both T2* and T1*ρ* need additional clarification^10,11^. To date, a solid body of evidence has been accumulated indicating the diagnostic potential of such qMRI techniques (excellently reviewed in^6,8,12^).

Even though previous studies have reported correlations between qMRI parameters on the one hand and structural as well as compositional features of the tissue on the other hand^13,14^, there is clearly no consensus on any specific relations between these parameters. Previously, our group studied correlations between measured qMRI parameter maps and modelled volume fractions to better refine each qMRI parameter’s sensitivity and specificity profile^15^. Additionally, quantitative T2 maps have been referenced to loading-induced changes in cartilage composition and structure based on a constitutive cartilage model^16^. However, to the best of our knowledge, multiparametric qMRI maps have not been modelled as a function of the functional properties of the tissue in general and its biomechanical measures in particular. Thus, this study aimed to establish a framework to integrate sample-specific multiparametric qMRI maps into the proposed constitutive material model while subsequently optimizing the model in terms of weighted structural and compositional tissue features as derived from the multiparametric qMRI maps. Therefore, the working hypotheses of the study were that (1) the complex relation between the stress response of the tissue and its qMRI appearance in terms of respective T1, T1*ρ*, T2 and T2* maps may be translated into a refined constitutive model of cartilage tissue and (2) the functional properties of the tissue can be described by this model following its comprehensive optimization.

## Results

Upon histological assessment, all samples were found to be grossly intact (Mankin Grade 0, mean sum score 3.2 ± 0.8).

### MRI measurements

**Figure 1:**
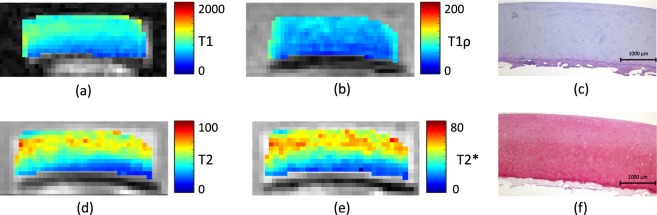
T1 (**a**), T1*ρ* (**b**), T2 (**d**) and T2* (**e**) maps of a representative histologically intact cartilage sample. Corresponding morphological images were used for the parameter map overlays. Histological evaluation in terms of Hematoxylin and eosin (**c**) as well as Safranin O staining (**f**) revealed no substantial structural or compositional tissue alterations. Units of scale bars are ms.

Spatially resolved quantitative T1, T2, T1*ρ* and T2* maps were obtained for all osteochondral samples. Qualitatively, samples were relatively homogeneous, while uniform changes in signal intensities were found as a function of tissue depth (Fig. 1). Whenever present, focal signal alterations were only slight, and, in any case, adjacent cartilage areas were not affected.

Quantitatively, qMRI parameter values were characterized by considerable standard deviations even though the mean parameter values were comparable. Table 1 presents a detailed overview of the qMRI parameter values.

**Table 1:** Quantitative characterization of human articular cartilage samples (n = 8) by qMRI parameters and the stiffness at a tissue strain of *ε* = 15%.

Sample No	T1 [ms]	T1*ρ* [ms]	T2 [ms]	T2* [ms]	Stiffness [MPa]	*R*^2^ [−]	Ω [%]
1	753.8 ± 184.4	50.6 ± 9	70 ± 10.9	39.7 ± 10.8	1.92	0.993	47
2	670.8 ± 101.1	37.1 ± 11.7	51.1 ± 13	32.6 ± 10.25	3.05	0.992	20
3	760.2 ± 207.8	56 ± 13.7	66.3 ± 15.2	47.5 ± 11.7	1.17	0.936	32
4	787.2 ± 72.9	47.4 ± 14.7	65.8 ± 13.5	37.4 ± 14	1.15	0.978	39
5	625.5 ± 145.2	35.1 ± 14.3	58.4 ± 15.4	27.1 ± 10.9	1.02	0.904	43
6	787.2 ± 120.7	46.6 ± 8.7	88.9 ± 31	37.6 ± 9.1	1.34	0.968	29
7	714.4 ± 156.4	46.5 ± 16.3	56.2 ± 13.2	38.7 ± 14.2	1.18	0.992	28
8	608.9 ± 105.5	28.3 ± 8.4	53.5 ± 11.4	24 ± 6.9	1.38	0.969	31
Mean ± SD	713.5 ± 42.35	44.4 ± 9.3	63.8 ± 11.4	35.6 ± 2.3	1.53 ± 0.68	0.966 ± 0.03	33 ± 9

The entire sample cross-sectional area was the region-of-interest. Data are given as mean ± standard deviation, while goodness-of-fit measures R^2^ and Ω detailing the correspondence between experimentally measured and theoretically modelled data are shown in the last two columns (from right).

### Confined compression tests

**Figure 2:**
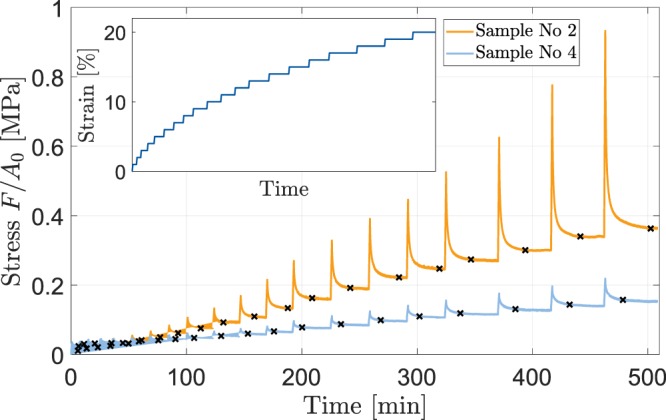
The confined compression stress response of two representative osteochondral samples as a function of time. Typical stress relaxation in response to sequential displacement-driven loading in the ramped configuration is observed. With higher strains, the constant strain phases were considerably extended to ensure proper tissue relaxation throughout the entire measurement period. Crosses indicate the convergence of the stress response at a relaxation rate of ≤0.03 N/min defined as the equilibrium state. The inset visualizes the applied loading protocol (i.e. strain [%] vs. time [min]).

The nominal stress response is illustrated in Fig. 2 as a function of time for two representative samples. Considerable variability in the stress responses of the samples can be seen. When keeping the sample strained, larger stress relaxation was observed with larger initial stresses.

**Figure 3:**
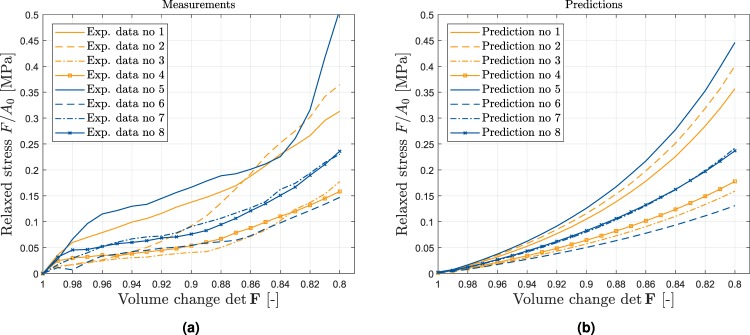
Relaxed nominal stresses (force per referential area) plotted versus intra-tissue volume changes (expressed by the the determinant of the deformation gradient det **F** = *J*). (**a**) Experimentally determined compression data. (**b**) Predictions on the basis of a computational model informed by sample-specific qMRI parameters. A detailed analysis of each sample’s correspondence of measured and modelled parameters is given in Table 1.

In Fig. 3a nominal stresses are plotted versus the intra-tissue volume changes. Similar to the stress-time response, the stress-induced volume changes were highly variable. Correspondingly, large standard deviations were found when calculating the mean stiffness at *ε* = 15%: *E* = 1.53 ± 0.68 MPa (mean ± SD). Sample-specific biomechanical testing details are displayed in Table 1.

### Model evaluation

Relaxed stress calculated by the model is plotted in Fig. 3b as stress versus intra-tissue volume changes. Predictions of the computational model (Fig. 3b) demonstrated grossly similar characteristics (in terms of trend and overall curve characteristics) as compared to the experimental measurements (Fig. 3a).

Quantitatively, the close correspondence of modelling-based predictions and experimental measurements was reflected by good-to-excellent goodness-of-fit measures: mean of *R*^2^: (M ± SD) = 0.966 ± 0.03, range (min, max) 0.904, 0.993; and mean of Ω = 33 ± 9%, range (min, max) 20%, 47% (Table 1).

**Figure 4:**
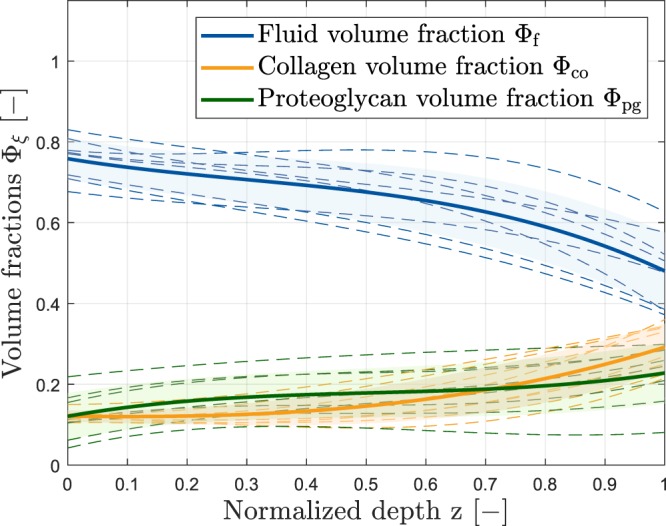
Volume fractions Φ_*ξ*_ (blue fluid, orange collagen, green proteoglycan) of the cartilage constituents as a function of normalized sample depth *z*. The solid bold line gives the mean volume fraction Φ_*ξ*_, while the dashed lines illustrate the sample-specific fractions and the shaded areas the standard deviations. Of note, the volume fractions are the result of qMRI measurements and each qMRI parameter’s weighting followed by comprehensive parameter optimization against the biomechanical measurements.

**Figure 5:**
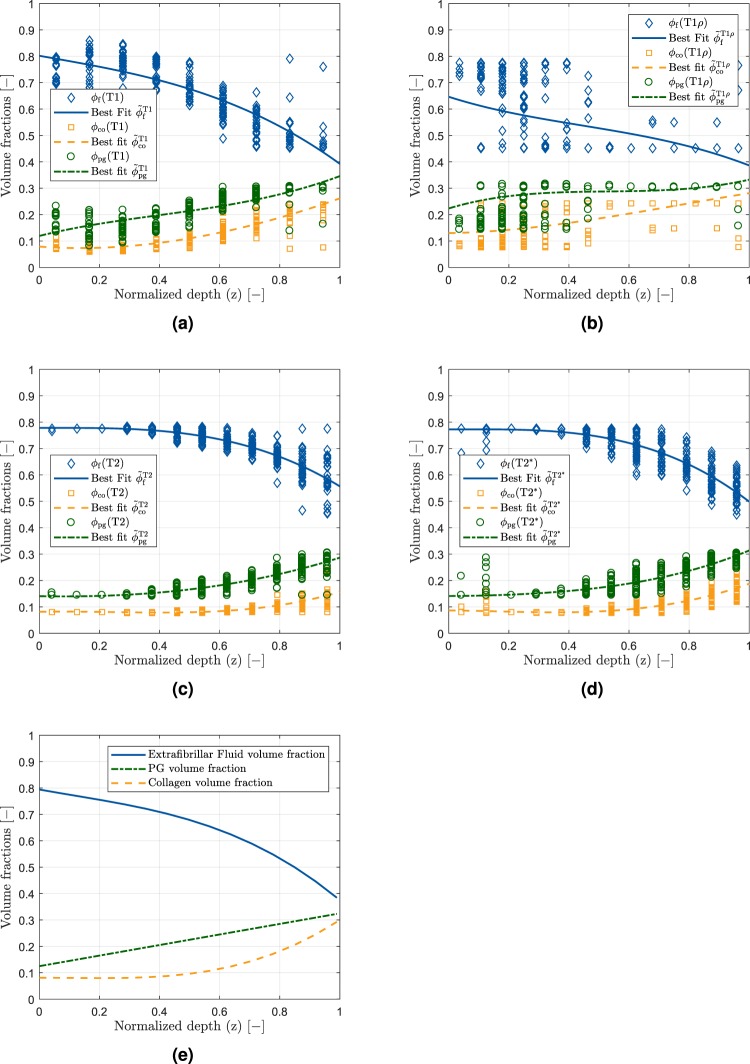
Optimization results for the one-dimensionally approximated spatial volume fractions ${\tilde{\varphi }}_{\xi }^{{\rm{T}}x}(z)$. Individual data points are indicated by checks (fluid), squares (collagen) and circles (proteoglycan) that demonstrate the *ϕ*_*ξ*_(T*x*) values of the qMRI parameters as a function of normalized sample depth. Solid lines represent the best-fit results. The data are shown for one representative sample for T1 (**a**), T1*ρ* (**b**), T2 (**c**) and T2* (**d**). For comparison, (**e**) illustrates the idealized model of the fractional distribution of fluid (solid, blue), proteoglycans (dashed-dotted, green) and collagen (dashed, orange) as a function of the normalized depth as proposed by Wilson *et al*.^23^.

The global set of material parameters obtained by the inverse Finite-Element optimization is given in Table 2. By assuming linear relations between the qMRI parameter-derived specific volume fractions ${\tilde{\varphi }}_{\xi }^{{\rm{T}}x}$ (*x* ∈ {1, 1*ρ*, 2, 2*}) and scalar coefficients ${w}_{{\rm{Tx}}}^{\xi }$, sample-specific volume fractions Φ_*ξ*_ for the fluid and collagen contents (*ξ* ∈ {f, co}) were obtained as a function of normalized sample depth *z* (see Fig. 4). Towards the sample surface, fluid content was high, while collagen and proteoglycan contents were low. Towards the sample bottom (i.e. the cartilage-bone transition), the differences in volume fractions were less pronounced even though fluid was still the dominant tissue component. Following optimization of the spatial volume fractions ${\tilde{\varphi }}_{\xi }^{{\rm{T}}x}(z)$ (Fig. 5a–d), the *ϕ*_*ξ*_(T*x*) values of the qMRI parameters showed close correspondence to the idealized model of the depth-related volume fractions as proposed by Wilson *et al*.^13^ (Fig. 5e). Accordingly, ${w}_{{\rm{Tx}}}^{\xi }$ represent weighting parameters which define each qMRI parameter’s contribution to the collagen and fluid volume fractions (please see MRI-based model input section below for more details). Additionally, six constitutive model parameters were obtained by the optimization procedure. Here, *k*_1_, *k*_3_ and *k*_3_ describe the material non-linearity of the collagenous part, while *a*_0_ and *a*_1_ are associated with the stiffness and compressibility of the non-collagenous matrix. *w* ∈ [0, 1] denotes a weighting parameter of the alignment of the collagen fibrils towards its preferred direction with the lower limit representing an ideal fibre alignment, while the upper limit expresses an isotropic fiber distribution.

**Table 2:** Details of the global material parameter set obtained by the inverse Finite-Element optimization of the spatial volume fractions in the framework of the computational model of cartilage.

	Weighting parameters				Material model parameters					
	${{\boldsymbol{w}}}_{{\bf{T1}}}^{{\boldsymbol{\xi }}}$	${{\boldsymbol{w}}}_{{\bf{T1}}{\boldsymbol{\rho }}}^{{\boldsymbol{\xi }}}$	${{\boldsymbol{w}}}_{{\bf{T2}}}^{{\boldsymbol{\xi }}}$	${{\boldsymbol{w}}}_{{\bf{T}}{{\bf{2}}}^{{\boldsymbol{\ast }}}}^{{\boldsymbol{\xi }}}$	*k*_1_	*k*_2_	*k*_3_	*a*_0_	*a*_1_	*w*
F (*ξ* = f)	0.41	0.42	0.16	0.01	0.6 MPa	50	25	0.35 MPa	5	0.3
CO (*ξ* = co)	0.31	0.12	0.11	0.46						

Weighting parameters (${w}_{{\rm{Tx}}}^{\xi }$) detail each qMRI parameter’s contribution to the collagen and fluid volume fractions and are to be read as follows: The fluid content of the tissue is primarily represented by T1 (41%) and T1*ρ* (42%), while the collagen content is primarily represented by T1 (31%) and T2* (46%). Six material model parameters complement the proposed constitutive model: *k*_1_, *k*_3_ and *k*_3_ are related to the biomechanical behaviour of collagen fibrils under tension, while *a*_0_ and *a*_1_ are associated with the stiffness and compressibility of the non-collagenous matrix. *w* describes the concentration of the collagen fibrils in their preferred direction.

## Discussion

The most important finding of this study is that sample-specific and spatially resolved qMRI data may be integrated into a computational model of cartilage to (1) emulate structural and compositional tissue parameters and to (2) reliably capture cartilage functional properties. The computational model of cartilage has been further refined and optimized to integrate imaging (i.e. the qMRI parameter maps) and biomechanical (i.e. its stress response to confined loading) information.

To the best of our knowledge, this is the first study to bring together imaging and functional tissue parameters within the framework of a computational model. QMRI data were used as input variables in a sample-specific manner, while the remaining parameters were kept constant in efforts to keep the model complexity manageable. In practical terms, the qMRI parameter maps were used to derive measures of structural and compositional tissue properties as a function of sample depth. To this end, the qMRI parameters have been weighted in their respective contributions to the tissue properties on the basis of an idealized cartilage model as proposed by Wilson *et al*.^17^. Since specific relations between the exact properties of the tissue and the qMRI parameters remain disputed and considerable overlap in specificity has been reported^10,15,16,18–20^ the first step was to identify weighting factors for the optimized qMRI-based tissue assessment.

In spite of the thorough and user-independent optimization against functional properties of the tissue, this framework can only provide the starting point to further study the correlations between imaging, compositional and biomechanical parameters. In particular, refined biochemical techniques providing spatially resolved measures of the tissue features such as microspectroscopy or polarized light microscopy (e.g.^21,22^) should be included in future studies to integrate sample-specific data on exact tissue properties rather than an idealized tissue model that gives tissue constituents as a function of depth. Nonetheless, the Wilson model of cartilage is validated and commonly used to convey depth-related information on tissue properties of cartilage^17,23–25^. Correspondingly, higher contents of extracellular fluid and lower contents of the solid constituents (i.e. collagen and proteoglycans) were found at superficial sample zones, while the opposite was observed for deeper cartilage zones. These compositional features are well in line with published reference data, e.g. by Wilson *et al*.^23^.

The computational model of cartilage was efficient enough to successfully describe biomechanical properties (e.g. stiffness) as obtained in the subsequent confined compression tests. Herein, both the non-linearity of the stress-relaxation tests as well as the biomechanical quantities determined at equilibrium (both measured and modelled) are reflective of earlier literature findings^26^. Even though the sample size of the present study is limited and the computational model of cartilage is not yet optimized in terms of refined compositional input variables (as outlined above), our results substantiate the fact that advanced qMRI techniques are adequate to determine relevant tissue features (in structure and composition) that determine the biomechanical properties and stress resilience of the tissue and may be used to non-invasively study tissue functionality. Once further optimized, the computational model might be used to complement clinical-standard MRI examinations to provide a detailed representation of the functional properties of the tissue and to identify tissue regions at risk of incipient degenerative changes. This is of particular relevance as articular cartilage is exquisitely sensitive to the mechanical environment and cartilage degeneration in OA is considered as the key pathophysiological result of abnormal mechanics^27,28^. In the clinical context, such patient-specific modelling approaches aim to obtain image-based surrogate parameters of tissue functionality to identify tissue areas at risk Nonetheless, these approaches need further refinement, in particular when translating the findings to the *in-vivo* and entire-joint configuration.

Although predictions of the proposed model were confirmed to a large extent by the experimental data, there was some discrepancy between modelled and measured datasets. Possible reasons involve a variety of as yet ill-controlled variables: Particular care was taken to standardize storage conditions, yet systematic error may have been introduced by the prolonged a storage in sample non-physiological environment. This may have led to alterations in the extracellular fluid content and biomechanical properties. Here, comparative longitudinal imaging-based assessment of cartilage functionality as a function of loading may be integrated into the model to further refine the input of data. Additionally, the distinct collagen network properties (in terms of orientation and integrity) have not been considered as additional input variables. In view of the ongoing scientific controversy on the imaging correlates of the collagen network^15,29^, future studies should take into account its complex features beyond its mere content, especially when the mechanical behaviour of the entire tissue is of interest. Here, the combination of mechanical stimuli and advanced qMRI techniques may help along since mechanical loading may be applied during imaging to study loading-induced intra-tissue changes to provide additional functional information. This can improve the computational model and its descriptive capacities^11,13,14,30^. Histology was used to confirm gross structural integrity of the cartilage tissue. However, samples may have been affected (in a functionally relevant context) beyond the histologically assessable scope, because OA is a disease that affects the entire joint by triggering catabolic and inflammatory processes in all compartments. As samples were harvested from total knee replacements only, our results remain to be confirmed in truly healthy cartilage tissue, e.g. from organ donor networks or tumor endoprothesis. Moreover, additional research activities have to be aimed at the inclusion of larger sample numbers of variable degeneration (as controlled by histology) to corroborate the potential of the model in predicting functional properties of cartilage in health and disease. Further limitations involve sample size and study setup. In this study, all measured data were used for thorough model parameter optimization. Larger sample sizes, including new samples, need to be included in future studies to assess the model’s predictive capabilities. To this end, the sample-specific qMRI data will be the only input data to the model, while the measured respective mechanical responses will be used as benchmark features. By applying k-fold cross validation schemes as in machine learning techniques (e.g.^31^) the model’s predictive capabilities can be assessed. Additionally, the confined compression tests used for biomechanical reference evaluation is unlike the actual weight bearing *in vivo*, thereby limiting our study’s transferability to the *in-vivo* setting. Against this background, more physiological forms of loading, quite possibly under simultaneous imaging^14,32^, should be applied in future studies.

In conclusion, this study introduces a computational model of cartilage that integrates qMRIN parameter maps as spatially resolved measures of the structural and compositional properties of the tissue to describe its biomechanical properties. On the basis of this model, advanced qMRI techniques may be complemented to comprehensively evaluate the functional dimension of non-invasively obtained imaging biomarkers. Thereby, cartilage degeneration (as the hallmark change of OA) may be appreciated in the context of abnormal mechanics and used as a potential target in diagnosing early (and potentially reversible) OA.

## Materials and Methods

### Study design

This study was designed as a prospective, comparative, intra-individual *ex-vivo* study that aimed to integrate the functional biomechanical properties of cartilage tissue and their qMRI correlates as input variables into the framework of a computational model of the tissue. Prior to this study, local Institutional Review Board approval from the Ethical Committee of RWTH Aachen University, Germany (AZ-EK157/13) was obtained. Only after individual oral and written informed patient consent was the material that had been collected intraoperatively included in the present study. Moreover, all consecutive experiments were performed in accordance with relevant guidelines and regulations.

### Cartilage sample preparation

Human articular osteochondral samples were prepared as in earlier studies^11,14,20^. Briefly, macroscopically intact osteochondral samples were obtained from eight consecutive patients undergoing total knee replacement at our institution from 11/2017 to 03/2018 (3 male, 5 female; age 64.5 ± 12.3 years [range, 40–77 years]). By obtaining one sample from one patient sample pooling was avoided. The osteochondral samples included in this study were harvested from patients with primary osteoarthritis. Therefore, all forms of secondary OA or other bone and joint disorders were excluded. After its sterile excision during surgery, the material was collected in Dulbecco’s modified Eagle’s medium (DMEM, Gibco-BRL, Gaithersburg, MD, USA) with a set of standard antibiotics added (i.e. 100 U/ml penicillin [Gibco], 100 *μ*g/ml gentamycin [Gibco] and 1.25 U/ml amphotericin B [Gibco]). The osteochondral samples were then prepared according to the standard procedure as follows: First, for the sake of topoanatomic consistency, samples were harvested from the lateral femoral condyles only. Second, samples were evaluated macroscopically according to the Outerbridge classification^33^ and only grossly intact samples were included (i.e. Outerbridge grades 0 and 1). Structural integrity was subsequently confirmed by means of histology (see below). Third, samples were cut to standard square shape (20 × 20 mm [width × length]) and the subchondral lamella was preserved, while all cancellous bone was removed. Samples had a mean thickness of 4.201 mm ±1.01 mm (Mean ± Standard Deviation). Using a rongeur, two notches were created at opposing sample sides to define the mid-sagittal plane for future reference.

### MRI measurements, data acquisition and analysis

After sample preparation, MR imaging examinations were performed on a per-sample basis using a clinical standard 3.0 T MRI scanner (Achieva, Philips, Best, The Netherlands). As before^11,30^, samples were placed in a transparent container fully immersed in DMEM solution with additives. Samples were imaged using a modified single-channel prostate coil (BPX-30 disposable endorectal coil, Medrad/ Bayer, Leverkusen, Germany)^14,20,34^. Particular attention was paid to position the samples at the iso-center of the coil while aligning the mid-sagittal plane along and the surface parallel to the main magnetic field B_0_. Prior to scanning, B_0_ inhomogeneities were excluded using B_0_ mapping. After scout views, proton-density weighted sequences were acquired in the axial, coronal and sagittal planes oriented perpendicular to each other (Table 3). On the basis of the axial views, the sagittal imaging section was guided along the mid-sagittal plane to generate a centrally bisecting plane through the sample. Afterwards, T1, T1*ρ*, T2, and T2* sequences were acquired with the sequence parameters detailed in Table 3. MR imaging was performed at room temperature monitored before and after the measurements (19.5 ± 0.7 °C). Once the data acquisition was completed, the MR raw data including time constants for each pixel were loaded into Matlab R2016a software (Natick, MA, USA). Then, spatially resolved parameter maps were generated by means of predefined and customized fitting routines on a per-pixel basis. Individual pixel values were determined with T1, T1*ρ*, T2 and T2* relaxation times calculated as follows

1 $${\rm{Signal}}(T\mathrm{1)}=|A[1-2\,\exp (\frac{-\,{T}_{I}}{T1})+\exp (\frac{-\,{T}_{R}}{T1})]|,$$

2 $${\rm{Signal}}({T}_{{\rm{S}}L})=A\exp (\frac{-\,{T}_{{\rm{S}}L}}{T1\rho }),$$

3 $${\rm{Signal}}({T}_{{\rm{E}}})=A\exp (\frac{-\,{T}_{{\rm{E}}}}{T2})+B,$$

4 $${\rm{Signal}}({T}_{{\rm{E}}})=A\,\exp (\frac{-\,{T}_{E}}{T{2}^{\ast }})\mathrm{.}$$

T1, T1*ρ*, T2 and T2* were the target relaxation times to be quantified on a per-pixel basis. Here, *T*_E_ is the echo time, T_SL_ the duration of the spin lock pulses, *T*_R_ the repetition time and *T*_I_ the inversion recovery time (i.e. the time delay between the initial inversion recovery pulse and the read out), while A and B are the signal pre-factor and offset, respectively, accounting for proton density and background noise. *R*^2^ statistics adjusted to the number of degrees of freedom was used to check the quality of the fits. For T2 and T2*, only pixel values of expected echo times (*T*_E_ ≤ 60 ms) were included to reduce the potential of mis-fitting. Sample segmentation was performed manually on the proton density-weighted morphologic images by choosing pixels that safely lay within the tissue. Boundary pixels were excluded to avoid partial volume effects. Segmentation of sample outlines was subsequently validated against the parameter overlays.

**Table 3:** Type, acquisition parameters and duration of the MR sequences.

Sequence	Type	Parameters	Duration [min:s]
PD	Turbospin echo	T_R_ 1500 ms; T_E_ 11.2 ms; ST 1 mm; SG 1.5 mm; 10 slices; AM 144 × 142; NSA 2; FOV 62 × 62 mm^2^; turbo factor 6, pixel size 0.43 mm	02:28
T1	Inversion recovery	T_R_ 3000 ms; T_E_ 10.1 ms; ST 2 mm; inversion times 150, 300, 500, 800, 1000, and 1500 msec; flip angle 90°; AM 224 × 220; NSA 1; FOV 62 × 62 mm^2^; turbo factor 5, pixel size 0.28 mm	09:09
T1*ρ*	Spin-lock multigradient echo	T_R_ 30 ms; T_E_ 3.83 ms; ST 3.2 mm; spin-lock durations 0, 10, 20, 30, 40 ms; flip angle 11°; AM 176 × 176; NSA 4; FOV 52 × 52 mm^2^; turbo factor 44, pixel size 0.3 mm	15:48
T2	Multispin echo	T_R_ 1500 ms; T_E_ = n * 8.38 ms (n = 1–12); ST 2 mm; flip angle 90°; AM 176 × 176; NSA 2; FOV 52 × 52 mm^2^; turbo factor 12, pixel size 0.3 mm	05:08
T2*	Multigradient echo	T_R_ 700 ms; T_E_ = 3.9 ms + n⋅ 9.6 ms (n = 1–14); ST 2 mm; flip angle 90°; AM 176 × 176; NSA 3; FOV 52 × 52 mm^2^; turbo factor 15, pixel size 0.3 mm	03:25

PD - Proton density. T_R_ - repetition time. T_E_ - echo time. ST - slice thickness. SG - interslice gap. AM - acquisition matrix. NSA - number of signal averages. FOV - field of view.

### Confined compression tests

**Figure 6:**
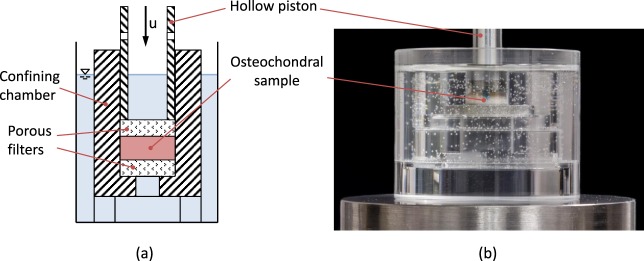
Schematic illustration (**a**) and photographic image (**b**) of the experimental test set-up used for confined compression. Displacement *u* was applied to the osteochondral sample through a hollow piston. The inner diameter of the confining chamber matched the outer diameter of both osteochondral sample and porous filters to provide exact sample confinement.

Within 24 h after the MR measurements, confined compression tests were conducted using a custom-made compression device. In line with literature data^26,35^, the osteochondral samples were placed in an impermeable confining chamber matching the dimensions of the samples (with a diameter of *d* = 8 *mm*). Porous stainless steel filters were placed above and underneath the samples to allow for fluid outflow. The samples were compressed by a hollow piston which axially moved down the upper filter (Fig. 6). The entire set-up was placed in a container filled with phosphate-buffered saline (PBS, Gibco) and mounted on a universal testing machine (Zwick Roell Z010, Zwick GmbH, Ulm, Germany).

Prior to the tests, a tare load of 1 N was applied to the osteochondral sample by lowering the piston at a rate of 0.16 mm/min to maintain standardized interaction of the interfaces throughout the measurements^26^. The resulting piston position was used to determine sample thickness, while equilibration was achieved by holding the piston in place for 15 min. Then, the osteochondral samples were subjected to a sequence of 20 ramped compressions with a strain-rate of 1%/min to a total strain of 20%. After each applied loading step, relaxation phases variable in duration (as depicted in Fig. 2) were applied to allow for sufficient equilibration^26^.

### Histological analyses

After biomechanical tests, samples underwent histological processing by simultaneous decalcification and fixation (Ossa fixona, Diagonal, Münster, Germany), sectioning along the mid-sagittal plane and embedding in paraffin. From the mid-sagittal plane, 5-*μ*m-thick sections were cut, stained with hematoxylin/eosin and Safranin O according to standard protocols and visualized using a Leica light microscope (model DM LM/P, Wetzlar, Germany). Histological grading was performed in a blinded manner using the Mankin classification^36^. Based on the quantitative assessment of tissue structure, cellularity, proteoglycan staining intensity and tidemark integrity, the Mankin sum score is a representative measure of tissue degeneration. Ranging from 0 to 14, a Mankin sum score of 0 is indicative of no histological signs of degeneration. Mankin sum scores may be grouped into distinct Mankin grades; i.e. Mankin grade 0 indicates structurally grossly intact cartilage (Mankin sum scores 0–4)^37^.

### MRI-based model input

The qMRI parameter maps were used as exclusive sample-specific input values within the finite element (FE) code. Material constants as well as the weighted qMRI parameters (see Table 2) were obtained in an inverse FE manner with an optimization algorithm updating the parameters for all samples simultaneously and thereby enforcing a global set of parameters.

Cartilage is classically considered as a bi-phasic material with a solid phase hydrated by interstitial fluid. The fluid-filled solid extracellular matrix primarily consists of the collagen (CO) fibril and proteoglycan (PG) fractions, in the following represented with respect to volume by *ϕ*_co_ and *ϕ*_pg_, respectively^17^. The interstitial fluid and solid volume fractions are denoted by *ϕ*_f_ and *ϕ*_s_, respectively. They are related by *ϕ*_s_ = 1 − *ϕ*_f_. The volume fractions are expressed as a function of the qMRI parameters T*x*, which satisfy the following condition:

5 $${\varphi }_{{\rm{pg}}}+{\varphi }_{{\rm{f}}}({\rm{T}}x)+{\varphi }_{{\rm{co}}}({\rm{T}}x)=\mathrm{1,}\,x\in \{\mathrm{1,\; 1}\rho {\mathrm{,\; 2,\; 2}}^{\ast }\}\mathrm{.}$$

Functional dependencies between qMRI parameters and idealized cartilage volume fractions as characterized in previous studies^15,16^ provided the basis for the qMRI-informed computational model of cartilage. More specifically, relations between the qMRI parameters and the fractional composition of an idealized cartilage model (as defined by Wilson *et al*.^23^) were created in a pixel-wise and depth-related manner for the fluid and CO fractions (*ϕ*_f_ and *ϕ*_co_, respectively). Note that this model of cartilage composition defines the mean content of each cartilage constituent as a function of tissue depth. These datasets served as the basis for the non-linear regression analysis with an exponential dependency ${\rm{T}}x({\varphi }_{\xi })=a\,\exp [b{\varphi }_{\xi }]+c$, where *a*, *b* and *c* are fitting coefficients. Based on earlier data^15,16^, these coefficients were determined by optimizing the contribution of each cartilage constituent to the qMRI parameter maps. Accordingly, the inverted generalized form is denoted by *ϕ*_*ξ*_(T*x*), where the function is continuously extended by the constant parameters *l*_T*x*,*ξ*_ and *u*_T*x*,*ξ*_ at the lower ${({\rm{T}}x)}_{\xi }^{{\rm{lo}}}$ and upper ${({\rm{T}}x)}_{\xi }^{{\rm{up}}}$ bound of definition.

Thus, one arrives at four distinct qMRI parameter specific volume fractions for CO and fluid, which were fitted against the pixel-wise resolved qMRI parameters. To this end, a one-dimensional phenomenological representation over the normalized depth coordinate *z* was employed as

6 $${\tilde{\varphi }}_{\xi }^{{\rm{T}}x}(z)=\frac{{\alpha }_{{\rm{T}}x,\xi }\,{z}^{3}+{\beta }_{{\rm{T}}x,\xi }\,{z}^{2}+{\gamma }_{{\rm{T}}x,\xi }\,z+{\delta }_{{\rm{T}}x,\xi }}{{\varepsilon }_{{\rm{T}}x,\xi }\,z+{\kappa }_{{\rm{T}}x,\xi }},\,\xi \in \{{\rm{co}},{\rm{f}}\},$$

where *α*_T*x*,*ξ*_, *β*_T*x*,*ξ*_, ..., *κ*_T*x*,*ξ*_ denote sample-specific constants obtained by optimization of the latter equation against the sample-specific qMRI data. A representative set of the fitting results is shown in Fig. 5.

It is accepted that no distinct qMRI parameter is specific for any particular cartilage constituent^8^. Thus, linearly weighted combinations need to be introduced to account for each qMRI parameter’s sensitivity and specificity profile. More specifically, scalar weighting factors of each constituent’s contribution were captured by ${w}_{{\rm{T}}x}^{\xi }$, while the respective cartilage constituent in relation to the qMRI parameter (T*x*) was determined by

7 $${{\rm{\Phi }}}_{\xi }(z)=\sum _{x}\,{w}_{{\rm{T}}x}^{\xi }\,{\tilde{\varphi }}_{\xi }^{{\rm{T}}x}(z),$$

where the normalization condition

8 $$\sum _{x}\,{w}_{{\rm{T}}x}^{\xi }=1$$

applies.

### Constitutive modeling

The deformation of cartilage was expressed by the deformation gradient **F** = Gradx, where ${\boldsymbol{x}}=\hat{{\boldsymbol{x}}}(X,t)$ denotes the current position vector.

A multiplicative decomposition of **F** into a volumetric part *J*^1/3^ and an isochoric (distortional) part $\bar{{\bf{F}}}$ was assumed^38^, where in *J* = det **F** denotes the relative volume change. The right Cauchy-Green tensor is given in terms of the deformation gradient as **C** = **F**^T^**F**, while its distortional part reads as $\bar{{\bf{C}}}={\bar{{\bf{F}}}}^{{\rm{T}}}\bar{{\bf{F}}}$.

As all cartilage tissue phases are considered incompressible, any volume decrease is assumed to be solely due to fluid outflow^39,40^. Hence, after complete fluid loss the fluid volume fraction is approximately 0 and the tissue volume approaches its solid constituent fraction (also referred to as compaction point at which *J* → Φ_*s*_)^41,42^. For a bi-phasic material the Helmholtz free energy (cf.^43^) can be given as

9 $${\rm{\Psi }}=\bar{{\rm{\Psi }}}(\bar{{\bf{C}}})+{{\rm{\Psi }}}_{\pi }(J,\mu )-\mu C,$$

where the first term on the right hand side ($\bar{{\rm{\Psi }}}(\bar{{\bf{C}}})$) reflects the isochoric free energy resulting from the deformation of the solid parts, while the second term (Ψ_*π*_(*J*, *μ*)) describes the chemo-mechanical interactions. The third term (*μC*) is determined by the chemical potential of the solvent *μ* and the molar solvent concentration $C=\frac{J}{{V}_{{\rm{m}}}}{{\rm{\Phi }}}_{{\rm{f}}}$ with the molar volume *V*_m_ (cf.^43,44^). Hence, *C* governs the fluid flux and is consequently neglected in the following, since the modelling is restricted to the relaxed (i.e. time-independent) configuration. Accordingly, the Cauchy stress tensor is given as $\sigma =2{J}^{-1}\,{\bf{F}}(\partial \bar{{\rm{\Psi }}}/\partial {\bf{C}}+\partial {{\rm{\Psi }}}_{\pi }/\partial {\bf{C}}){{\bf{F}}}^{-{\rm{T}}}$, where^45^

10 $$\frac{\partial \bar{{\rm{\Psi }}}}{\partial {\bf{C}}}={J}^{-\mathrm{2/3}}[\bar{{\bf{S}}}-\frac{1}{3}(\bar{{\bf{S}}}:{\bf{C}}){{\bf{C}}}^{-1}],\,\frac{\partial {{\rm{\Psi }}}_{\pi }(J)}{\partial {\bf{C}}}=-\,\pi J{{\bf{C}}}^{-1},\,\bar{{\bf{S}}}=\frac{\partial \bar{{\rm{\Psi }}}}{\partial \bar{{\bf{C}}}},$$

while $\bar{{\bf{S}}}$ represents the isochoric contribution of the second Piola-Kirchhoff stress tensor. Furthermore, *π* denotes the osmotic multiplier and can be given by an empiric expression (cf.^43^) as

11 $$\pi =-\,\frac{\partial {{\rm{\Psi }}}_{{\rm{x}}}}{\partial J}={a}_{0}{(\frac{1-{{\rm{\Phi }}}_{{\rm{s}}}}{J-{{\rm{\Phi }}}_{{\rm{s}}}})}^{2{\tilde{a}}_{1}}+{\pi }_{0},\,{\tilde{a}}_{1}={{\rm{\Phi }}}_{{\rm{f}}}\,{a}_{1}\mathrm{.}$$

(11)_2_ is a phenomenological relation enforcing a stronger correlation with the sample-specific qMRI data. *a*_0_ and *a*_1_ are material parameters, while *π*_0_ ensures a stress-free reference configuration. As the extracellular matrix is composed of the solid cartilage phase (i.e. CO and PG)^23,39,40^, its free energy can be given as

12 $$\bar{{\rm{\Psi }}}={{\rm{\Phi }}}_{{\rm{c}}{\rm{o}}}\,{\bar{{\rm{\Psi }}}}_{{\rm{c}}{\rm{o}}}(\bar{{\bf{C}}},{\bf{L}})+{{\rm{\Phi }}}_{{\rm{p}}{\rm{g}}}\,{\bar{{\rm{\Psi }}}}_{{\rm{p}}{\rm{g}}}(\bar{{\bf{C}}}),$$

where ${\bar{{\rm{\Psi }}}}_{{\rm{co}}}(\bar{{\bf{C}}},{\bf{L}})$ represents the anisotropic contribution of the CO fibril network and ${\bar{{\rm{\Psi }}}}_{{\rm{pg}}}(\bar{{\bf{C}}})$ is the free energy contribution of the isotropic non-collagenous matrix. The isotropic part of (12) is modelled by the Neo-Hookean model as ${\bar{{\rm{\Psi }}}}_{{\rm{pg}}}={a}_{0}({\bar{I}}_{1}-3)$ with the parameter *a*_0_ which enforces a stress-free reference configuration, see (11)_1_. ${\bar{I}}_{1}={\rm{tr}}\bar{{\bf{C}}}$ denotes the first isochoric strain invariant. The fibril network is modelled by a polyconvex expression which can be given as (cf.^46,47^)

13 $$\begin{array}{rcl}{\bar{{\rm{\Psi }}}}_{{\rm{c}}o} & = & \sum _{i}^{n}\,{k}_{1}[f({\bar{I}}_{4i})+g({\bar{K}}_{i})]\\  &  & =\,\sum _{i}^{n}\,{k}_{1}[\frac{1}{{\tilde{k}}_{2}}\exp \{{\tilde{k}}_{2}({\bar{I}}_{4i}-1)\}+\frac{1}{{\tilde{k}}_{3}}({\bar{K}}_{i}^{{\tilde{k}}_{3}}-1)],\,{\tilde{k}}_{\eta }={{\rm{\Phi }}}_{{\rm{co}}}\,{k}_{\eta },\,\eta \in \{2,3\}\mathrm{.}\end{array}$$

Here, the exponential part $\mathrm{1/}{\tilde{k}}_{2}\,\exp \{{\tilde{k}}_{2}({\bar{I}}_{4i}-1)\}$ captures the J-shape response to tension typical for CO fibres, while the latter term $1/{\tilde{k}}_{3}({\bar{K}}_{i}^{{\tilde{k}}_{3}}-1)$ describes the fibre contribution to the tissue response to compression due to the tube contraction effect^48,49^. *k*_1_, *k*_2_ and *k*_3_ denote material constants. The local CO fibril architecture is largely responsible for cartilage anisotropy. Hence, in the constitutive model, cartilage anisotropy is captured by weighted structural tensors **L**_*i*_ and associated structural invariants ${\bar{I}}_{4i}$ and ${\bar{K}}_{i}$ (*i* = 1, 2, …, *m*) defined by^47,50^

14 $${\tilde{{\bf{L}}}}_{i}=\frac{{w}_{i}}{3}{\bf{I}}+\mathrm{(1}-{w}_{i}){{\bf{L}}}_{i},\,{{\bf{L}}}_{i}={{\rm{m}}}_{i}\otimes {{\rm{m}}}_{i},\,{\bar{I}}_{4i}={\rm{tr}}(\bar{{\bf{C}}}{{\bf{L}}}_{i}),\,{\bar{K}}_{i}={\rm{tr}}[({\rm{cof}}\,\bar{{\bf{C}}}){\tilde{{\bf{L}}}}_{i}],$$

where *w*_*i*_ (*i* = 1, 2, ..., *m*) denote scalar weighting parameters associated with the preferred fibre families whose directions are specified by unit vectors ***m***_*i*_. **I** denotes the identity tensor. Finally, in view of (10)_3_ and (12), the isochoric part of the second Piola-Kirchhoff stress tensor can be given as

15 $$\bar{{\bf{S}}}={{\rm{\Phi }}}_{{\rm{co}}}\,\sum _{i}^{m}\,{k}_{1}[\{\,f^{\prime} ({\bar{I}}_{4i})-g^{\prime} ({\bar{K}}_{i})\}{\tilde{{\bf{L}}}}_{i}+g^{\prime} ({\bar{K}}_{i}){\bar{K}}_{i}\,{\bf{I}}]+{{\rm{\Phi }}}_{{\rm{pg}}}\,{a}_{0}\,{\bf{I}}.$$

### Numerical implementation and parameter estimation

For the parameter identification, the computational model of cartilage proposed in the previous section was implemented as a user-subroutine UMAT (user material routine) into the commercially available software package Abaqus FEA (v6.17, Simulia Corp., Providence, USA). To emulate the idealized Arcade model of Benninghoff ^51^, the preferred fibril directions *θ*_fib_(*z*) were implemented in a rotationally symmetric manner with eight equiangular fibre families as before^16^. The weighted fractional relations (7) were mapped onto the sample-specific geometry. To exclude the possibility of negative volume fractions as a result of the saturation condition (5), the condition of minimally positive PG fraction values was enforced by the tolerance value Δtol = 1 × 10^−6^.

Algorithm 1 presents a detailed overview of the code used for FE implementation of the computational model of cartilage. In the confined compression test the sample-specific geometry was implemented on the basis of its mean thickness, while the piston was modeled as a rigid body.

The parameter fitting was performed using a constrained optimization in Matlab while satisfying condition (8). The minimal error between the FE simulations and actual experiments for the complete set of samples (n = 8) served as the objective function. An overview of the optimization framework can be found in Algorithm 2.

The accuracy of the simulations as compared to the experimental measurements was quantified by determining the square of Pearson’s correlation coefficient (i.e. *R*^2^ as in^52^) and the relative approximated error defined by ${\rm{\Omega }}=100\cdot \Vert ({\boldsymbol{y}}-{\boldsymbol{f}})\Vert /\Vert {\boldsymbol{y}}\Vert ,$ where ***y*** and ***f*** denote vectors of experimental and computational values, respectively.

**Algorithm 1:**
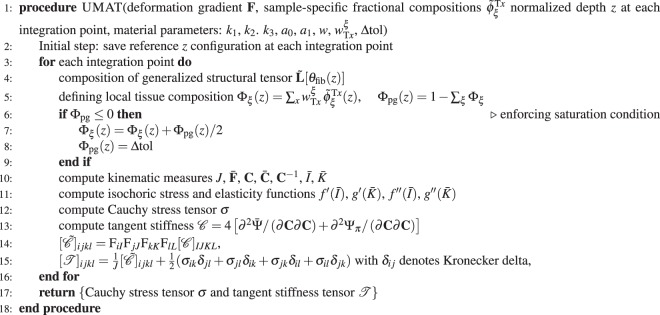
Finite-element implementation of the material model informed by multiparametric qMRI data. Prior to computation, the compositional functions defining the contribution of each qMRI parameter to the structural and compositional properties of the tissue have to be derived. Please see section “Constitutive modelling” for more details.

**Algorithm 2:**
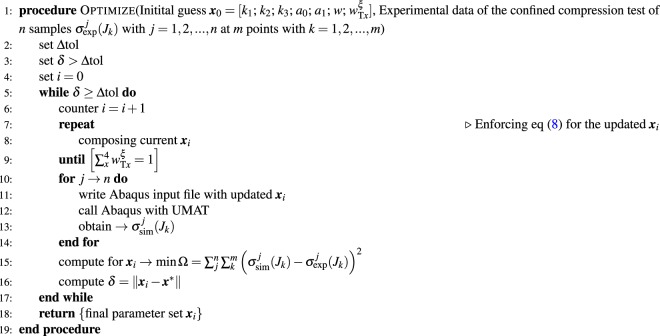
Optimization of material constants as well as qMRI parameter weightings. x* denotes a local minimum.

## Data Availability

All data generated or analysed during this study are included in this published article
